# The Synergistic Effects of Aminosilane Coupling Agent on the Adhesion Performance of Silane Primer for Silicone Resin Thermal Protection Coating

**DOI:** 10.3390/polym15102361

**Published:** 2023-05-18

**Authors:** Ting Pan, Zhenhua Su, Yue Yan, Xiaofei Zhu, Fan Qi, Lianbin Wu

**Affiliations:** 1 Key Laboratory of Organosilicon Chemistry and Material Technology Ministry of Education, Key Laboratory of Organosilicon Material Technology of Zhejiang Province, College of Material Chemistry and Chemical Engineering, Hangzhou Normal University, Hangzhou 311121, Chinasuzhenhua_szh@icloud.com (Z.S.);; 2 Shanghai Aerospace Chemical Application Institute, Huzhou 313002, China

**Keywords:** aminosilane coupling agent, synergistic effects, silane primer, adhesion property, silicone resin thermal protection coating

## Abstract

As a bridge between the coating and the substrate, the primer has a direct impact on the adhesion performance of silicone resin thermal protection coating. In this paper, the synergistic effects of an aminosilane coupling agent on the adhesion performance of silane primer were investigated. The results show that silane primer containing N-aminoethyl-3-aminopropylmethyl-dimethoxysilane (HD-103) formed a continuous and uniform film on the surface of the substrate. Two amino groups of HD-103 were conducive to moderate and uniform hydrolysis of the silane primer system, and the introduction of dimethoxy groups was more conducive to the improvement of interfacial layer density and the formation of the planar surface structure, thus enhancing the bond strength at the interface. When the content was 13 wt%, it exhibited excellent synergistic effects on adhesive properties, and the adhesive strength reached 1.53 MPa. The possible morphology and composition of the silane primer layer were investigated by scanning electron microscopy (SEM) and X-ray photoelectron spectroscopy (XPS). A thermogravimetric infrared spectrometer (TGA-IR) was used to analyze the thermal decomposition of the silane primer layer. The results showed that the alkoxy groups in the silane primer were first hydrolyzed to form Si-OH, and then the dehydration and condensation reactions between Si-OH and the substrate formed a firm network structure.

## 1. Introduction

Due to aerodynamic heat, the high-speed aircraft surface will be exposed to extreme thermal shock during the launch and flight of the aircraft, which imposes strict requirements on the materials of the corresponding parts [1,2,3]. Thermal protection coating technology is used for coatings that must withstand high temperatures and maintain their integrity and good physical and mechanical properties for long periods at high temperatures [4,5,6]. The coatings can be ablated at ultra-high temperature to form a dense protective layer, with good thermal insulation and small bulk density [7]. As an important organic–inorganic hybrid resin, silicone resin has been widely used in the design and application of thermal protection materials [8]. The silicone resin-based thermal protection composites provide high temperature resistance not only through the composite material itself but also through the interface with the protective substrate [9]. Therefore, how to achieve effective adhesion of silicone resin thermal protection coatings to the substrates has become a major challenge limiting their application.

The primer is a solution-like substance that is pre-coated on the adherend during the adhesive construction process and forms a paint film after curing, which plays a vital role in the interface adhesive [10,11,12]. The components of the primer include the film former, cross-linking tackifier, catalyst, and diluent, among which the film former and the cross-linking tackifier play a major role [13,14]. At present, there are many types of film former, including silicone [15,16], epoxy resin [17,18], polyurethane, acrylic resin [19,20], urethane, etc. Silane coupling agents as cross-linking tackifiers are the main components used for crosslinking and thickening [21,22,23]. Silane-based primers are widely used in the tackifying treatment of high-temperature-resistant resin-based composite materials [24] and substrates [25]. Toorani et al., used tetraethylorthosilicate and 3-aminopropyltriethoxysilane silane for silanization of the coating on the surface of AZ31B Mg alloy, and the results showed an increase in the corrosion resistance of the coating [26]. Grard et al. used silane and reactive polysiloxane to silanize the surface of AA6061 and enhanced the adhesion between a high consistency silicone rubber (HCR) and AA6061, improving its protective properties [27]. Angelja et al., used bis-(3-(3-(3-triethoxysilyl)propyl) thioureido)propyl-terminated polydimethylsiloxane to improve the protective properties of a coating on AA2024 Al alloy with similar results [28]. Among silane coupling agents, an agent known to have a general structural formula is (RO)3-Si-Y (Y represents organic functional group with amino groups; OR represents alkoxy groups); it can provide active sites for bonding different interfaces and shows great potential on the application of primers. Hirotaka et al. successfully introduced amino groups into cellulose paper by in situ modification. The amino groups introduced into cellulose paper can act as an efficient alkaline catalyst to improve the catalytic efficiency and reusability of the paper [29]. However, the effects of aminosilane coupling agents on the hydrolysis and curing behavior, including the adhesion performance of silane primers used for thermal protection coating, have not been thoroughly or systematically studied [30].

Herein, according to the outlined facts, the synergistic effects of aminosilane coupling agents on the adhesive performance between the substrate and the silicone resin thermal protective coating were studied by SEM, XPS, TGA-IR, and FT-IR. This contribution will provide a theoretical basis to guide the development of silane primers for silicone thermal protective coatings.

## 2. Experiment

### 2.1. Materials

The epoxy primer and silicone resin thermal protection coating were supplied by Shanghai Institute of Aerospace Power Technology. Tetraethylorthosilicate (TEOS, AR) was supplied by Sinopharm Co., Ltd., Shanghai, China; Propylorthosilicate (TPOS, 97%), 3-aminopropyltriethoxysilane (APTES, 99.5%) were supplied by Sass Chemical Technology Co., Ltd., Washington, DC, USA; Butylorthosilicate (TBOS, 95.0%) was supplied by Bide Pharmaceutical Technology Co., Ltd., Shanghai, China; Tetrabutyl titanate (Ti(OBu)_4_, 99.0%) was supplied by Macklin Biochemical Co., Ltd., Shanghai, China; 2-butoxy ethanol (C_6_H_14_O_2_, 98.0%) was supplied by Wuxi Zhanwang Chemical Reagent Co., Ltd., Yixing City, China; 3-aminopropyltrimethoxysilane (APTMS, 97.0%), Diethylenetriaminopropyltrimethoxysilane (NQ-62, AR) were supplied by Aladdin Biochemical Technology Co., Ltd., Shanghai, China; 3-(2-aminoethyl)-aminopropyltrimethoxysilane (DAMO, 97.0%) was supplied by Shanghai Energy Chemical Co., Ltd., Shanghai, China; N-aminoethyl-3-aminopropylmethyl dimethoxysilane (HD-103, 96.0%) was supplied by Aladdin Biochemical Technology Co., Ltd.; KD-504A adhesive was supplied by Ningbo Tiandong Adhesive Co., Ltd., Ningbo, China; Gasoline was supplied by Shenzhen Baodi Chemical Co., Ltd., Shenzhen City, China; distilled water was self-prepared.

### 2.2. Preparation and Application of Silane Primer

**Scheme 1:**
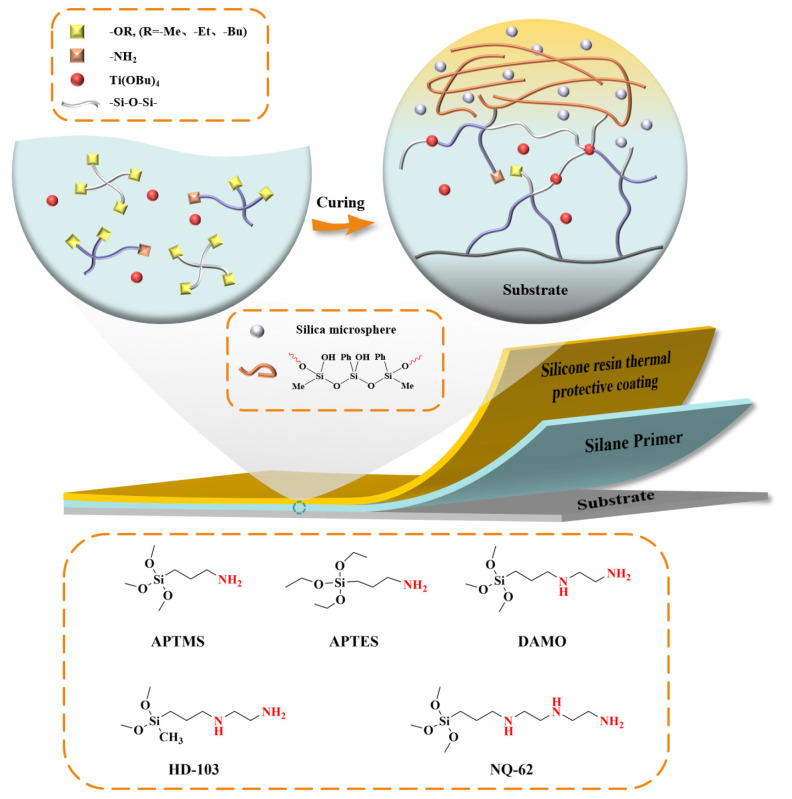
Diagram of thermal protective coating structure.

To apply the silane primer layer on the substrate, using Ti(OBu)_4_ and 2-butoxy ethanol as catalysts and solvents, respectively, five different aminosilane coupling agents (Scheme 1) in gasoline solutions of TEOS, TPOS, and TBOS with a ratio of 1:3:1 were prepared. The obtained silane primers were named P-tetraalkoxysilanes (as blank), P-APTMS, P-APTES, P-DAMO, P-HD103, and P-NQ62. Before spraying silane primer, the tinplate coated with epoxy primer was sanded with sandpaper (the tinplate coated with epoxy primer was used as the substrate in this study); then, the surface of the substrate was wiped with ethyl acetate. The silane primer was evenly sprayed on the substrate and cured in a constant temperature and humidity box set at 35 °C and 50% RH (relative humidity) for 60 min. Next, silicone resin thermal protective coatings were sprayed on the silane-coated samples. Finally, the thermal protective coating was cured in a 40 °C drying oven for 1 day and cured in the air for 5 days. The thermal protective coating diagram is shown in Scheme 1.

### 2.3. Characterization

The tensile shear strength of the thermal protective coating applied to the samples treated with various silane primers were obtained by a material testing machine using British Lloyd Company LS-100. KD-540A type AB glue (component A: component B = 1:2) was used to bond the two samples; the bonding area was 2 cm × 2 cm. Samples were stored at room temperature for 2 days to ensure that the glue was completely cured. The adhesion properties of the silane primer were tested according to GB/T7124-2008. The material testing machine was pulled at a speed of 100 mm/min until the coatings on the two samples were separated from each other. All tests were performed on five samples and averaged.

The micromorphology was observed by scanning electron microscope (SEM, HITACHI Sigma-500, Hitachi, Tokyo, Japan) at room temperature using HITACHI Sigma-500. The substrates were evenly cut into 5 mm × 5 mm squares and sprayed with silane primer by spray gun at a concentration of 0.4 mL/cm^2^. The unsprayed samples and the samples after spraying for a specific time were metalized with a thin gold layer before observation. 

X-ray photoelectron spectroscopy (XPS, Thermo Scientific ESCALAB Xi+, Thermo Fisher Scientific, Waltham, MA, USA) was used to detect radicals after curing of the silane primer on the substrate surface. The silane primer was sprayed on the 1 cm × 1 cm substrate surface with a spray gun, and XPS energy spectrum scanning was performed after the silane primer was cured.

A thermogravimetric infrared spectrometer (TGA-IR, Netzsch STA449F3-Bruker VERTEX 70, Netasch, Germany) was heated from 35 °C to 800 °C at a heating rate of 10 °C/min in a nitrogen atmosphere. The heat resistance and thermal decomposition of the cured silane primer were explored, and the generated gas was analyzed by IR to assist in the analysis of the hydrolysis and curing mechanism of the silane primer.

The hydrolysis performance of the silane primer was observed by Fourier transform infrared spectroscopy (FT-IR, Thermo Fisher Scientific, Waltham, MA, USA) using the Thermo Nicolet 5700. Due to the low concentration of the active ingredients of the original silane primer, the performance was difficult to observe after hydrolysis and solidification. Therefore, the concentration of the silane primer was increased to 25% of the original volume and dropped quantitatively on copper paper, and the infrared spectrum was measured at a specific time.

## 3. Results and Discussion

### 3.1. Adhesion Properties

Since tetraalkoxysilane is the main film former in the silane primer, the effect of the tetraalkoxysilane ratio on the adhesion performance of the silane primer was investigated. As shown in Table 1, Compared with A, E, and F, when only one tetraalkoxysilane existed, TPOS had best performance in adhesion properties, while TEOS was similar to TBOS. The effect of different ratios of TEOS, TPOS, and TBOS was investigated on the adhesion properties. When the ratio of the three silanes was 1:3:1, the adhesive strength of the silane primer was the highest (1.33 MPa). The silane primer was accompanied by the evaporation of alcohol during the hydrolysis curing process [31], and the SEM results of Figure S1 (Supplementary Materials) show that during the alcohol evaporation process, a flat film structure gradually formed on the substrate surface, which was conducive to the formation of a thin film of silane primer [5], with a thickness of 1.83 μm (Figure S2). The effect of the amount of TPOS on the bonding performance was also studied. With the increase of the TPOS content, the adhesive strength showed a trend of first increasing and then decreasing (H-K in Table 1). It can be seen that excessive TPOS was not conducive to the improvement of the interfacial bonding strength of silane primer.

**Figure 1:**
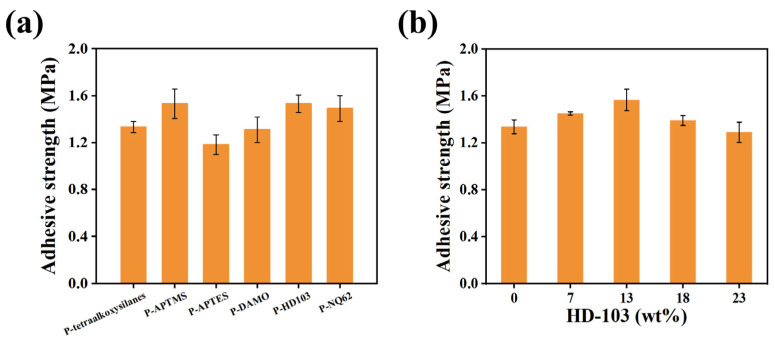
Adhesion strength of (**a**) P-tetraalkoxysilane, P-APTMS, P-APTES, P-DAMO, P-HD103, P-NQ62 and (**b**) silane primer with different HD-103 concentrations.

To study the synergistic influence of aminosilane coupling agents on the adhesive properties of silane primer, five aminosilane coupling agents were selected and introduced into the tetraalkoxysilane primer at a concentration of 13 wt%. The results were shown in Figure 1a, where the adhesive strength of P-tetraalkoxysilanes was 1.33 MPa and it can be seen that the adhesion performance of P-APTES and P-DAMO silane primer decreased. For P-DAMO, the easily hydrolyzed methoxy group grew in a spatial network structure during the hydrolysis process. In addition, two amino groups gave P-DAMO a stronger catalytic effect than APTES, which contained one amino group. An excessively fast hydrolysis rate leads to asynchronous hydrolysis of the film-forming agent and tackifier, reducing the density of cross-links and resulting in the cross-links not being in the same plane. The adhesive strengths of the other three silane primers were enhanced to varying degrees. The catalytic effect of the three amino groups in NQ-62 led to an excessively fast hydrolysis rate, so that the film-forming agent ended the hydrolysis and condensation prematurely when sufficient cross-linking points were not formed at the interface. In contrast, the hydrolysis of the three methoxy groups in APTMS was slow and sufficient due to the low catalytic efficiency, resulting in the formation of a large number of cross-linking points at the interface to enhance the adhesive strength. In addition, the two amino groups contained in HD-103 can promote the hydrolysis of the film-forming agent in the silane primer and condense with the methoxy group, forming a flat network structure at the interface to increase the cross-linking point. APTMS and HD-103 provided a positive effect on the film-forming property of the silane primer, and the adhesion performance was improved more obviously. Hence, HD-103 was used for subsequent research.

The effect of HD-103 content on the adhesion performance of silane primers was further investigated. As shown in Figure 1b, the adhesive strength of the silane primer increased as the content of HD-103 increased. The maximum adhesive strength occurred when the concentration of HD-103 was 13 wt% and thereafter decreased with the increasing of HD-103 concentration. The catalytic effect of the 7 wt% HD-103 silane primer system was weak and could not promote complete hydrolysis of the film-forming agent in the silane primer to form more cross-linking points. This was similar to the number of cross-linking points generated at the interface when the 0 wt% HD-103 silane primer was hydrolyzed; thus, the improvement of the adhesive strength was not obvious. On the contrary, the excessive amino group brought by 23 wt% HD-103 silane primer caused too-fast catalytic efficiency, resulting in an overly rapid hydrolysis and condensation rate of the film-forming agent; thus, the number of cross-linking points generated at the interface decreased, resulting in the decrease in adhesive performance. However, a mild catalytic rate was conducive to the slow and uniform hydrolysis of 13 wt% and 18 wt% HD-103 silane primer, which in turn increased the number of interface cross-linking points and greatly enhanced the adhesive strength at the interface. Due to the addition of HD-103, the adhesive strength of the silane primer was slightly higher than that of P-APTMS, and the error range was also smaller than the latter. In addition, the hydrolytic group in P-HD103 contained only two methoxy groups, which allowed the direction of hydrolytic condensation to grow towards a plane, which was more conducive to improving the adhesive strength of the primer. Therefore, the following studies were carried out with 13 wt% HD-103 silane primer.

### 3.2. Surface Morphology of Silane Primer

**Figure 2:**
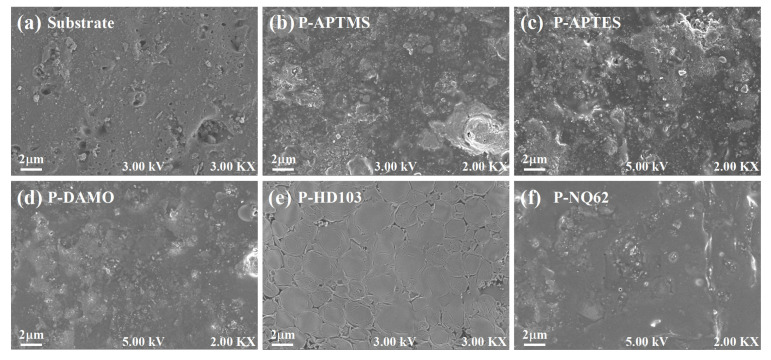
SEM of (**a**) the substrate and (**b**) P-APTMS; (**c**) P-APTES; (**d**) P-DAMO; (**e**) P-HD103; (**f**) P-NQ62 after curing for 60 min.

**Figure 3:**
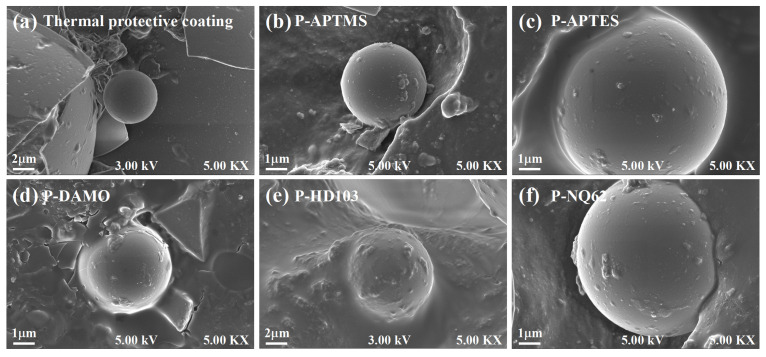
SEM of (**a**) thermal protective coating surface and (**b**) P-APTMS; (**c**) P-APTES; (**d**) P-DAMO; (**e**) P-HD103; (**f**) P-NQ62 after curing for 60 min.

The number of amino groups for aminosilane coupling agents will have an impact on the cross-linked network structure formed and the rate of hydrolysis and curing. Thus, the microscopic morphology of the silane primer containing five aminosilane coupling agents on the substrate and thermal protective coating was observed by SEM to explore the curing process. It can be clearly observed from Figure 2 and Figure 3 that they formed a film covering the surface pores on the surface of the substrate, while certain agglomerates were formed on the silica microspheres on the surface of thermal protection coating. From Figure 2b,c, it can be seen that a large number of agglomerates were formed on the surface of the substrate after curing of P-APTMS and P-APTES. Compared with P-APTMS, the agglomerates formed by the P-APTES after curing were larger. From Figure 2b,d,f, it can be observed that the increase in the number of amino groups in the side chain of the aminosilane coupling agent led to an enhanced catalytic effect, which made the hydrolysis and curing reaction more thorough. This resulted in fewer block agglomerates after curing and therefore a more uniform film, whereas the thickness of the final film decreased with increasing amino groups. It can be observed from the Figure 2d,e that P-HD103 formed a uniform film on the surface of the substrate, while P-DAMO was agglomerated. This was due to the difference in the number of alkoxy groups, leading to the formation of an ordered planar network structure in the former, while a disordered spatial network structure was formed in the latter. At the same time, it can be observed in Figure 3 that only P-HD103 could eventually form a thin film on the surface of the silica microspheres, while other silane primers failed to form a film but formed aggregates of different degrees. This confirmed the importance of the number of alkoxy groups in film formation.

**Figure 4:**
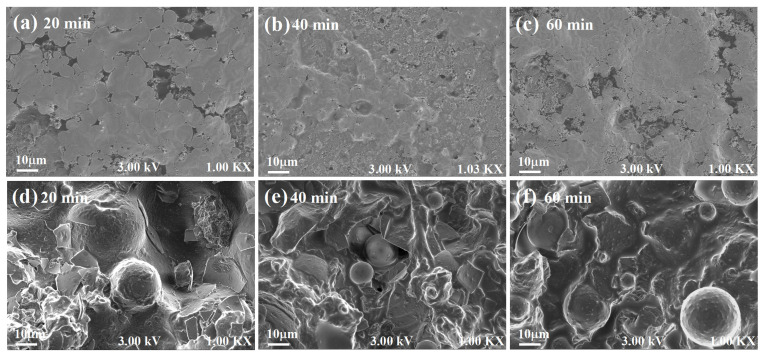
SEM of P-HD103 cured on substrate surface for (**a**) 20 min, (**b**) 40 min, and (**c**) 60 min and cured on thermal protective coating surface for (**d**) 20 min, (**e**) 40 min, and (**f**) 60 min.

The microscopic surface structure formed by the hydrolysis of the silane primer on the substrate was one of the key factors that affected the adhesive performance. In general, uneven and discontinuous surfaces displayed poor adhesive performance under harsh conditions, such as mechanical abrasion and corrosive solution environments. In order to further explain the hydrolysis and curing process of the silane primer, firstly, microscopic images of P-Tetraalkoxysilane with time on the surface of the substrate were observed by SEM (Figure S1a–c). From Figure S1, it can be seen that a thin film composed of nano-scale silica aggregates was formed on the surface of the substrate after 20 min of curing. Within 20–40 min, the film developed further, which resulted in fewer cracks on the surface of the film. In the following 20 min, the film grew further, forming a small number of agglomerates. These nano-sized silicas were formed by the hydrolysis and curing of tetraalkoxysilane in the silane primer. Due to the lack of aminosilane coupling agent (crosslinking tackifier), the silica nanoparticles failed to grow in a certain direction during the hydrolysis and curing process, and the aggregated silica nanoparticles failed to form a dense film structure. According to the above results, P-HD103 had the best adhesive effect among the five aminosilane coupling agents. Thus, SEM was used to analyze the hydrolysis and curing process (Figure 4a–c). Within the first 20 min of the reaction, the silane primer formed a discontinuous film covering the surface of the substrate; after 20 min, the presence of HD-103 promoted the hydrolytic cross-linking between silanes, making the surface film appear smooth and continuous; from 40–60 min, the hydrolysis and crosslinking between the films continued to form a thicker and denser silane layer. It can be seen that the aminosilane coupling agent can act as a tackifier to promote hydrolysis and crosslinking, and the two methoxy groups in HD-103 can help to form a uniform and flat silane primer layer.

Figure 4d–f shows the curing process of P-HD103 on the surface of the thermal protective coating: within the first 40 min, the film formed by the curing of the silane primer gradually changed from uneven and irregular to smooth and continuous; after 40–60 min, the silane primer was further hydrolyzed and cured to form a thicker film that covered the whole surface of the thermal protection coating. Compared with the hydrolysis process of the silane primer without the aminosilane coupling agent (Figure S1d–f), the curing of the silane primer containing HD-103 first produced a film structure and then further formed nano-silica particles, while the former was directly cured to form nano-silica particles. As a result, a mild catalytic environment and an appropriate amount of macromolecules could facilitate the hydrolysis of silane to form a relatively flat membrane structure rather than an inhomogeneous aggregate.

### 3.3. Surface Composition of Silane Primer

**Figure 5:**
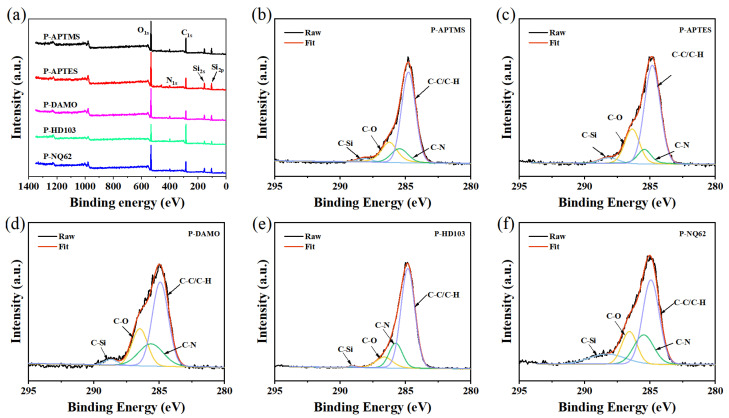
(**a**) XPS survey total spectra and C1s single spectra of (**b**) P−APTMS, (**c**) P−APTES, (**d**) P−DAMO, (**e**) P−HD103, (**f**) P−NQ62 silane primer on the substrate.

The XPS survey spectra of the silane primer on the substrate surface are shown in Figure 5. In order to determine elemental composition and the chemical state of the elements, the single spectra of the silane primer are shown as well. The survey spectrum shows the characteristic peaks of C, Si, and N. Figure 5 shows the C1s measured spectra of five silane primers after curing on the substrate surface. The peak with the most intensity at binding energies of 284.80 eV corresponds to C–C or C–H, referring to the NIST (National Institute of Standards and Technology) database. The peaks located at binding energies of 288.15 eV, 286.59 eV, and 285.47 eV are attributed to C–Si, C–O, and C–N respectively.

C1s showed the comparison of the chemical bond content of the five silane primers after curing, which mainly showed the difference of C-O bonds. P-HD103 had the lowest number of C–O bonds, which was due to its more thorough hydrolysis and higher degree of curing than other silane primers. However, P-APTES, P-DAMO, and P-NQ62 all contained more C–O bonds because there remained more unreacted alkoxysilanes after hydrolysis and curing, which was consistent with our previous results. Meanwhile, the N1s signal was curve-fitted into two different peaks (Figure S3); the peaks at 401.15 and 399.24 eV corresponded to the N-H and N-C bonds, respectively.

### 3.4. Thermal Stability of Silane Primer

**Figure 6:**
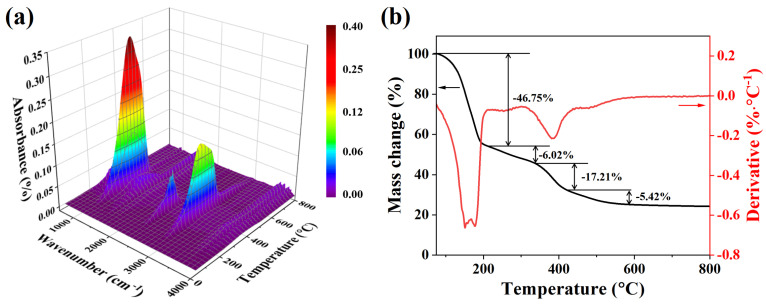
(**a**) TGA-IR and (**b**) TGA curve of P−HD103.

In order to explore the thermal degradation process of the silane primer, TGA-IR measurements were used to analyze the evolved gas products in nitrogen. Figure 6a displays the 3D TGA-IR spectra of the evolved gas in the thermal degradation of P-HD103. There were three distinct absorption bands with multiple absorption peaks at 3000–2815 cm^−1^, 2400–2260 cm^−1^, and 1200–980 cm^−1^, which were assigned to the absorption peaks of C-H, CO_2_, and CO, as well as the low boiling point TEOS and alcohols (refer to Figure S4). With the increase of temperature, the peak intensity was significantly enhanced, and two different degrees of thermal weight loss can be observed in the corresponding TGA diagram (Figure 6b), which are attributed to the further hydrolysis of the silane primer and the incompletely reacted TPOS and TBOS (the boiling points are 225 °C and 277 °C, respectively) generated during the curing process, accompanied by the degradation and carbonization of the alkoxy groups in the silane primer. When the pyrolysis temperature exceeded 400 °C, the CO_2_ absorption peak disappeared, and the alkoxy groups continued to condense to produce a large amount of alcohol. When the temperature reached 600 °C, the volatile groups in the silane primer were basically completely decomposed, and the final residual mass percent remained at 24.6% due to the stability of the Si–O–Si skeleton.

### 3.5. The Hydrolysis and Curing of the Silane Primer on the Substrate

**Figure 7:**
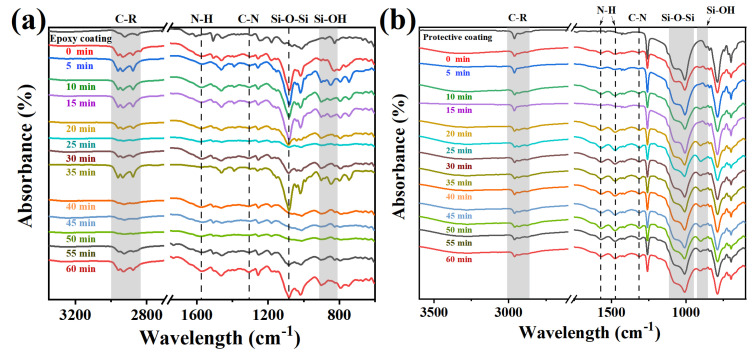
Infrared spectrum of (**a**) substrate and (**b**) thermal protective coating sprayed with P-HD103 and cured within 60 min.

Figure 7a shows the spectra for P-HD103 every 5 min for 60 min on the substrate surface. From 0–20 min, the film-forming agent tetraalkoxysilane in the silane primer was hydrolyzed, and the absorption peak of Si–OH appeared at 800–850 cm^−1^, accompanied by the production of Si–O–Si. At the same time, the N–H in the film former HD-103 in the silane primer appeared at 1240 cm^−1^ and 1460 cm^−1^, indicating that HD-103 had begun to work and appeared on the surface. From 20–40 min, hydrolysis further occurred, and the hydrolysis and condensation reaction of the tackifier and the film-forming agent in the silane primer proceeded at the same time. The tetraalkoxysilane in the whole system was basically hydrolyzed into Si–OH with a single absorption peak, and Si–OH was further condensed to form Si–O–Si with the SiO_2_ structure of Si–O–Si gradually increasing. However, the change of Si−O−Si peak shape was hardly observed in the infrared from last 40 min to 60 min stage. In the 40 min to 60 min stage, the hydrolysis and condensation were basically completed; there was also Si–OH compounded on the surface in addition to a large number of Si–O–Si structured compounds. Meanwhile, the amino functional group of HD-103 remained on the surface, which could further catalyze the condensation reaction of Si–OH with other oxide groups to form a strong Si–O–Si bond. At the same time, the self-curing of P-HD103 also showed similar curing processes and results (as shown in Figure S5).

Figure 7b shows the curing process of P-HD103 on the surface of the thermal protective coating substrate. Since the coating was a silicone resin thermal protection coating, the Si–O–Si structure produced during the surface curing process was not obvious in the infrared spectrum. It can be observed from Figure 7b that the gasoline solvent had been completely volatilized initially. At 0–20 min, a weaker multiplet of Si–OH was produced at 800 cm^−1^–900 cm^−1^ due to hydrolysis, which had a small number of compounds but a large number of species. At the same time, a Si–O–Si absorption peak appeared at 1000 cm^−1^–1200 cm^−1^ due to the condensation of Si–OH. At 20–40 min, the film-forming agent in the silane primer was further hydrolyzed, so that the absorption peak of Si–OH changed to a weak single absorption peak, and the presence of HD-103 at this time could even continue to promote the hydrolysis and curing. From 40 to 60 min, hydrolysis and condensation presented a dynamic process and continued to form Si–O–Si structural compounds.

## 4. Conclusions

In summary, TPOS played the most important role as a binder in the silane primer, and the best bond strength was achieved when the ratio of TEOS:TPOS:TBOS was 1:3:1. Among the five aminosilane coupling agents, HD-103 exhibited the best synergistic effect on the adhesive performance due to its mild catalytic effect and the formation of a unique planar network structure on the interface. XPS and FT-IR results showed the hydrolytic curing behavior of the silane primer and the final curing results: the siloxane in the silane primer was first hydrolyzed to Si–OH and then dehydrated and condensed between Si–OH to form a Si–O–Si network structure.

## Figures and Tables

**Table 1:** Adhesive strength of silane primer with different tetraalkoxysilane ratio.

Sample	TEOS	TPOS	TBOS	Tensile Shear Strength (MPa)	Failure Mode
A	3	0	0	0.843	Interface damage
B	2	1	0	0.852	Interface damage
C	0	1	2	0.875	Interface damage
D	0	2	1	0.912	Interface damage
E	0	0	3	0.820	Interface damage
F	0	3	0	0.973	Interface damage
G	1	2	0	0.974	Interface damage
H	1	2	1	1.138	Interface damage
I	1	3	1	1.330	Interface damage
J	1	4	1	0.937	Interface damage
K	1	5	1	0.668	Interface damage

## Data Availability

Not applicable.

## Supplementary Materials

The following supporting information can be downloaded at: https://www.mdpi.com/article/10.3390/polym15102361/s1, Figure S1: SEM of P-tetraalkoxysilanes cured on substrate surface (a) 20 min; (b) 40 min; (c) 60 min and cured on thermal protective coating surface (d) 20 min; (e) 40 min; (f) 60 min. Figure S2: SEM of the cross-section of P−tetraalkoxysilane after 60 min of curing. Figure S3: XPS survey spectra: N1s single spectra of primers on the substrate. Figure S4: Characterization of heat resistance of P−HD103: IR absorbance curve of pyrolysis product as a function of temperature. Figure S5: The infrared spectrum of HD-103 primer cured within 60 min.
