# Supplementary material for: The Synergistic Effects of Aminosilane Coupling Agent on the Adhesion Performance of Silane Primer for Silicone Resin Thermal Protection Coating
Source: Polymers (Basel). 2023 May 18;15(10):2361. doi: 10.3390/polym15102361 (PMC10220930; doi:10.3390/polym15102361)
Supplement: Supplementary file 1 [file polymers-15-02361-s001.zip › polymers-2380143-supplementary.pdf]

Supplementary Materials

The Synergistic Effects of Aminosilane Coupling Agent on the Adhesion Performance of Silane Primer for Silicone Resin Thermal Protection Coating

Ting Pan [1,†], Zhenhua Su [1,†], Yue Yan [1], Xiaofei Zhu [2], Fan Qi [1,*] and Lianbin Wu [1,*]

1 Key Laboratory of Organosilicon Chemistry and Material Technology Ministry of Education, Key Laboratory of Organosilicon Material Technology of Zhejiang Province, College of Material Chemistry and Chemical Engineering, Hangzhou Normal University, Hangzhou 311121, China; panting024@163.com (T.P.); suzhenhua_szh@icloud.com (Z.S.); maesyan@hznu.edu.cn (Y.Y.)
2 Shanghai Aerospace Chemical Application Institute, Huzhou 313002, China; huxiaofei@fudan.edu.cn
* Correspondence: eleven@hznu.edu.cn (F.Q.); wulianbin@hznu.edu.cn (L.W.)
† These authors contributed equally to this work.

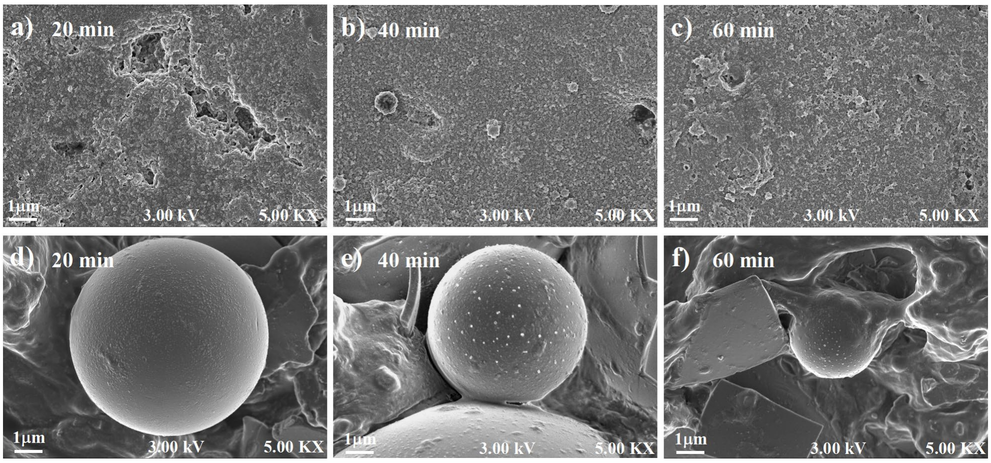

Figure S1. SEM of P-tetraalkoxysilanes cured on substrate surface (a) 20 min; (b) 40 min; (c) 60 min and cured on thermal protective coating surface (d) 20 min; (e) 40 min; (f) 60 min.

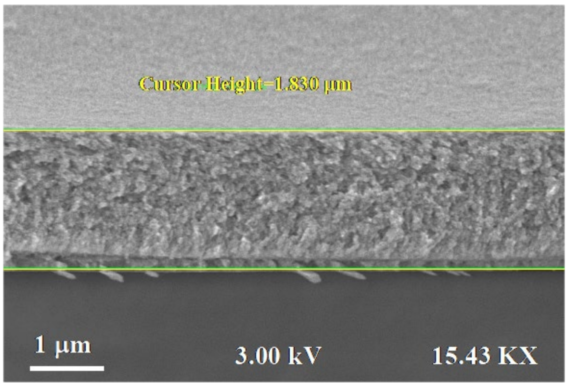

Figure S2. SEM of the cross-section of P-tetraalkoxysilane after 60 min of curing.

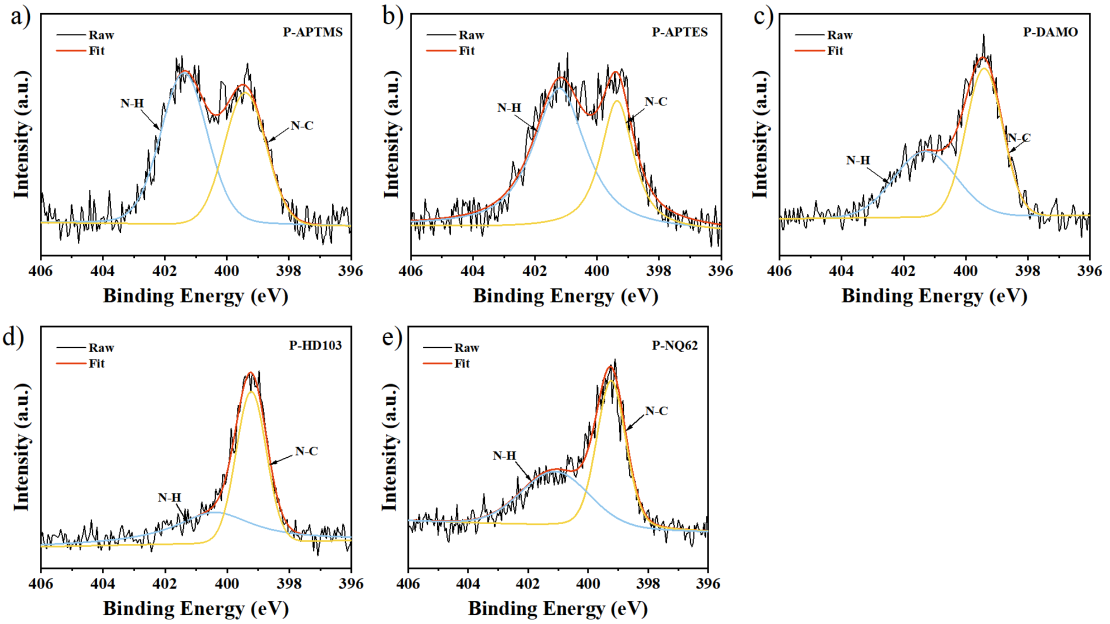

Figure S3. XPS survey spectra: N1s single spectra of primers on the substrate.

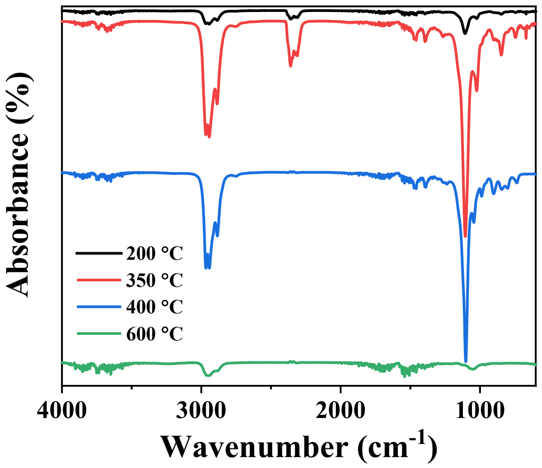

Figure S4. Characterization of heat resistance of P-HD103: IR absorbance curve of pyrolysis product as a function of temperature.

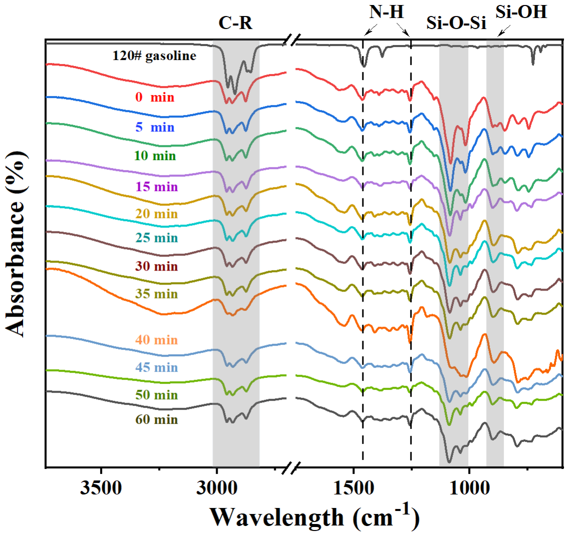

Figure S5. The infrared spectrum of HD-103 primer cured within 60 min.
